# Treatment of Protrusion of the Lower Teeth

**Published:** 1898-03

**Authors:** J. N. Farrar

**Affiliations:** New York.


ARTICLE VIII.

### TREATMENT OF PROTRUSION OF THE LOWER TEETH.

BY DR. J. N. FARRAR, NEW YORK.

Anterior displacement of the lower law resulting from loss of side teeth (thus permitting the two jaws to approach nearer each other than is normal) causing the front lower teeth to slide, scissors-like, upon the labial surfaces of the upper teeth, may sometimes be corrected by so grinding the front teeth that a part of the ends of these teeth will occlude and gradually slide each other into their proper relations. When the abnormality, however, is caused by disproportion in the amount of alveolar tissue constituting the alveolar ridge, or by two short side teeth causing a difference between the places of the antagonizing ends of the side and front teeth, it is found that the lower ones are generally found so far anterior to the upper that grinding would not often prove beneficial. If the difference between the planes of the arches is too great to correct by grinding, the teeth of the two jaws should be removed in the directions that will cause the best outlines of the face, and then force the proper occlusion by grinding the interfering parts.

For correcting a displaced lower jaw one of the best plans is by the use of gold thimble crowns, cemented upon the side teeth; the heading of the thimbles being so inclined that by occlusion the lower jaw will tend to slide posteriorly.

In cases where the upper front teeth require to be moved forward and outward it may be done by causing absorption, or by springing the outer plates of the sockets outward until the best possible contour of the upper lip is accomplished. The object of this is not only to place the front upper and lower teeth in better relation to each other, but, if possible, to move anteriorly and make more prominent the bony foundation of the upper part of the sunken upper lip and the sunken alæ of the nose. This latter change, moving of the bone, however, cannot always be accomplished without injury to some anterior tooth. This arises from too great strain upon the lateral incisors. In order to form the best outline or contour of both lips in such cases, it is sometimes best, all things considered, to let the lower lip fall a short distance, by moving posteriorly the lower front teeth. When there are no missing side-teeth, it is sometimes necessary to extract one tooth on each side to permit the moving of the front teeth posteriorly. Occasionally, however, extraction of one of the incisors (lower) and closing the space thus made by forcing postertorly the others will suffice for this end. To bring about the highest degree of facial beauty cannot always be done by following strictly the rules laid down by sculptors, where there is abundance of material and opportunity to obtain any effect desired; but the aim should always be to obtain the highest possibilities toward harmony of all the features that the conditions of the case may permit.

### PROTRUDING LOWER AND RECEDING UPPER TEETH CORRECTED BY AN INCLINED PLANE.

To show the value of an inclined plane, the case of a girl about thirteen years of age is given. Her side teeth, molars, cuspids and bicuspids, were very short, and although the roots were not fully developed, the side teeth had erupted sufficiently to occlude properly. The lower incisors, however, were more than an inch anterior to their proper places and occluded anterior to the upper incisors, the wrong contact causing both the upper and lower teeth to continue to move each other still farther out of place. The lower lip, as a consequence, was of course too prominent, and the upper one not sufficiently so.

The patient could by effort draw back the lower jaw so as to permit the edges of the upper and lower incisors to occlude, but could not draw it sufficiently back to enable the lower incisors to occlude posterior to the upper ones.

All that was necessary to give the short side teeth an opportunity to further erupt, and at the same time force the lower incisors backward and the upper ones forward, was to wear an inclined plane on the lower teeth. This plane was beveled to such an acute angle that all the incisors, upper and lower, moved at the same time oppositely. *Dental News.*

---

ARTICLE IX.

